# The genetic and pharmacogenomic landscape of snoRNAs in human cancer

**DOI:** 10.1186/s12943-020-01228-z

**Published:** 2020-06-23

**Authors:** Yaoming Liu, Hang Ruan, Shengli Li, Youqiong Ye, Wei Hong, Jing Gong, Zhao Zhang, Ying Jing, Xiulan Zhang, Lixia Diao, Leng Han

**Affiliations:** 1 Department of Biochemistry and Molecular Biology, McGovern Medical School at The University of Texas Health Science Center at Houston, Houston, TX 77030 USA; 2 State Key Laboratory of Ophthalmology, Zhongshan Ophthalmic Center, Sun Yat-sen University, Guangzhou, 510623 People’s Republic of China; 3 Hubei Key Laboratory of Agricultural Bioinformatics, College of Informatics, Huazhong Agricultural University, Wuhan, 430070 TX People’s Republic of China; 4 Department of Bioinformatics and Computational Biology, The University of Texas MD Anderson Cancer Center, Houston, TX 77030 USA

**Keywords:** Small nucleolar RNA, Genetic variants, Pharmacogenomics, Cancer

## Abstract

Emerging evidence has revealed significant roles for small nucleolar RNAs (snoRNAs) in tumorigenesis. However, the genetic and pharmacogenomic landscape of snoRNAs has not been characterized. Using the genotype and snoRNA expression data from The Cancer Genome Atlas, we characterized the effects of genetic variants on snoRNAs across 29 cancer types and further linked related alleles with patient survival as well as genome-wide association study risk loci. Furthermore, we characterized the impact of snoRNA expression on drug response in patients to facilitate the clinical utility of snoRNAs in cancer. We also developed a user-friendly data resource, GPSno (http://hanlab.uth.edu/GPSno), with multiple modules for researchers to visualize, browse, and download multi-dimensional data. Our study provides a comprehensive genetic and pharmacogenomic landscape of snoRNAs, which will shed light on future clinical considerations for the development of snoRNA-based targeted therapies.

## Main text

Small nucleolar RNAs (snoRNAs) are a group of regulatory RNAs that mainly reside in the nucleolus and post-transcriptionally modify ribosomal RNA (rRNA) and small nuclear RNA (snRNA). Emerging evidence has demonstrated significant roles of snoRNAs in cancer [1]. For example, deletion of SNORD50A/B may cooperate with oncogenic KRAS mutations in cancer to drive Ras-MAPK hyperactivation [2]. Overexpression of SNORD46 has been shown to promote cancer cell growth, migration, and invasion [3]. Recent studies characterized the expression landscape and copy number variations of snoRNAs in large numbers of cancer patients [2, 3]. It remains challenging to further understand the functional roles of snoRNAs in cancer. For example, it is unclear whether genetic variants will affect the expression level of snoRNAs, and whether the alterations of snoRNAs are associated with drug response in patients.

In this study, we investigated the effects of genetic variants on snoRNA expression and characterized the effects of snoRNA expression on drug response. To facilitate broad access of these data for the biomedical research community, we developed a user-friendly database, GPSno. We expect this study to have a significant clinical impact on the future development of snoRNA-based targeted therapies.

## Results and discussions

### Impact of genetic variants on snoRNA expression

**Fig. 1:**
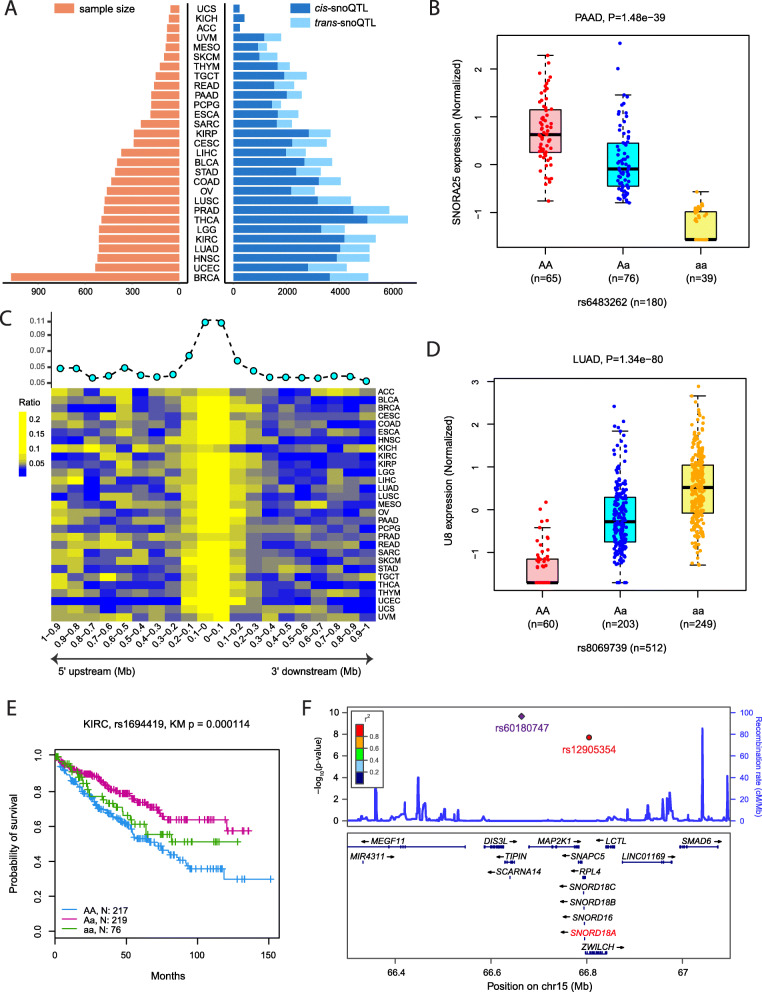
Summary of pan-cancer snoQTL analysis. **a** Numbers of samples included and numbers of snoQTLs identified in different cancer types. **b** Association between *cis-*snoQTL rs6483262 alleles and SNORA25 levels in PAAD. **c** Relative location distribution of *cis-*snoQTLs in regard to their paired snoRNAs. **d** Association between *trans-*snoQTL rs8069739 alleles and U8 levels in LUAD. **e** Kaplan–Meier plot displaying the association between rs1694419 genotypes and overall survival times of KIRC patients. **f** snoQTL rs12905354 located in TCGT GWAS locus

To comprehensively characterize the impact of genetic variants on snoRNA expression across different cancer types, we performed snoRNA expression quantitative trait loci (QTL) analysis [4] across 29 cancer types with at least 50 patients having both genotype data and snoRNA expression data in The Cancer Genome Atlas (TCGA) (additional file: Fig. S1A and Table S1). A total of 9449 tumor samples were included, with the sample size of each cancer type ranging from 56 in uterine carcinosarcoma (UCS) to 1073 in breast invasive carcinoma (BRCA) (Fig.1a). After imputation and quality control for the genotype data, we obtained a median of 4,358,817 SNPs for each cancer type. There were on average 435 snoRNAs (reads per kilobase per million reads [RPKM] ≥1) for each cancer type (additional file: Table S2). For analysis of proximal genetic regulation of snoRNAs (SNP within 1 Mb from the snoRNA location), a total of 69,557 significant SNP–snoRNA pairs in 29 cancer types were identified at a per-tissue false discovery rate (FDR) < 0.05 (Fig. 1a), which corresponded to a median *P*-value < 8.34 × 10^− 9^. The number of *cis*-snoQTLs ranged from 227 in UCS to 5067 in thyroid carcinoma (THCA), with a median of 2184 *cis*-snoQTLs per cancer type (Fig. 1a; additional file: Table S2). For example, in pancreatic adenocarcinoma (PAAD), rs6483262 alleles demonstrated significant effects on regulating the expression of SNORA25 (*P*-value = 1.48 × 10^− 39^) (Fig. 1b). To be noted, SNORA25 was reported as a promising biomarker for the early detection of pancreatic cancer [5]. We also examined the relative location distribution of *cis-*snoQTLs in regard to paired snoRNAs, and found that *cis-*snoQTLs were preferentially located in proximity to paired snoRNAs (Fig. 1c). For analysis of the remote genetic regulation of snoRNAs (SNP beyond 1 Mb from the snoRNA location), a total of 34,151 SNP–snoRNA pairs in 29 cancer types were identified at a per-tissue FDR < 0.05 (Fig. 1a), which corresponded to a median *P*-value < 7.40 × 10^− 15^. The number of *trans*-snoQTLs ranged from 0 in adrenocortical carcinoma (ACC) to 1525 in THCA, with a median of 821 *trans*-snoQTLs per cancer type (Fig. 1a; additional file: Table S2). For example, rs8069739 alleles showed remote regulation of the expression of U8 in lung adenocarcinoma (LUAD) (Fig. 1d). It was reported that U8 depletion triggers a remarkably potent p53-dependent anti-tumor stress response in lung adenocarcinoma [6]. The numbers of *cis*-snoQTLs and *trans*-snoQTLs were both significantly correlated with the number of samples (Spearman’s correlation, *cis*-snoQTL: Rs = 0.92, *P*-value = 3.48 × 10^− 12^; *trans*-snoQTL: Rs = 0.87, *P*-value = 6.52 × 10^− 10^) (additional file: Fig. S1B). This suggests that the effects of some SNPs may be underestimated due to insufficient sample size in certain cancer types, and this could be improved with additional patient samples in the future.

To investigate the clinical relevance of these genetic variants associated with snoRNA expression, we identified 475 SNP alleles that correlated with patients’ overall survival times in different cancer types with FDR < 0.05. For instance, kidney renal clear cell carcinoma (KIRC) patients carrying the homozygous rs1694419 AA had worse overall survival than those carrying the heterozygous Aa and the homozygote aa (log-rank test, *P*-value = 1.14 × 10^− 4^) (Fig. 1e). Patients carrying the homozygous rs1694419 AA had significantly higher expression level of SNORD45B than patients carrying the heterozygous Aa and the homozygous aa in 26 out of 29 (89.7%) cancer types, including KIRC (*P*-value = 3.93 × 10^− 34^, additional file: Fig. S2A). SNORD45B was significantly upregulated in KIRC tumor tissues compared to adjacent normal tissues (Student’s t-test, *P*-value = 0.011; additional file: Fig. S2B), and KIRC patients with higher expression of SNORD45B also had worse overall survival than those with lower expression of SNORD45B (log-rank test, *P*-value = 0.0041; additional file: Fig. S2C). Taken together, our results demonstrated the effects of snoQTLs on patients’ survival through regulating the expression of snoRNAs.

To identify genome-wide association study (GWAS)-related snoQTLs, 28,345 trait/disease-related SNPs were extracted from the GWAS catalog, and 1,167,961 SNPs were obtained that were located in the GWAS linkage disequilibrium (LD) regions. We identified 29,795 snoQTL–snoRNA pairs in which the snoQTLs overlapped with known disease/trait-associated loci in different cancer types. For example, the Testicular Cancer Consortium found that rs60180747 is significantly associated with testicular germ cell tumor (TGCT) risk (odds ratio [OR] = 1.23; *P* = 1.10 × 10^− 10^) [7]. The SNP marker rs60180747 marks a 261 kb haploblock on 15q22.31 that contains several genes, including *TIPIN, MAP 2 K1*, *DIS3L, SNAPC5, RPL4* and *ZWILCH* (Fig. 1f), and rs60180747 is located in the intron of gene *TIPIN*. However, this risk allele is not correlated with any protein-coding genes in 15q22.31 in TGCT patients [8]. We further examined nearby SNPs, and observed that rs12905354, which is in LD with rs60180747 (LD r^2^ = 1.0), significantly correlated with the expression of SNORD18A (additional file: Fig. S3) rather than protein-coding genes located in this risk locus. These findings suggest that SNORD18A may be a causal target in this TGCT GWAS loci.

### Pharmacogenomic landscape of snoRNAs across different cancer types

**Fig. 2:**
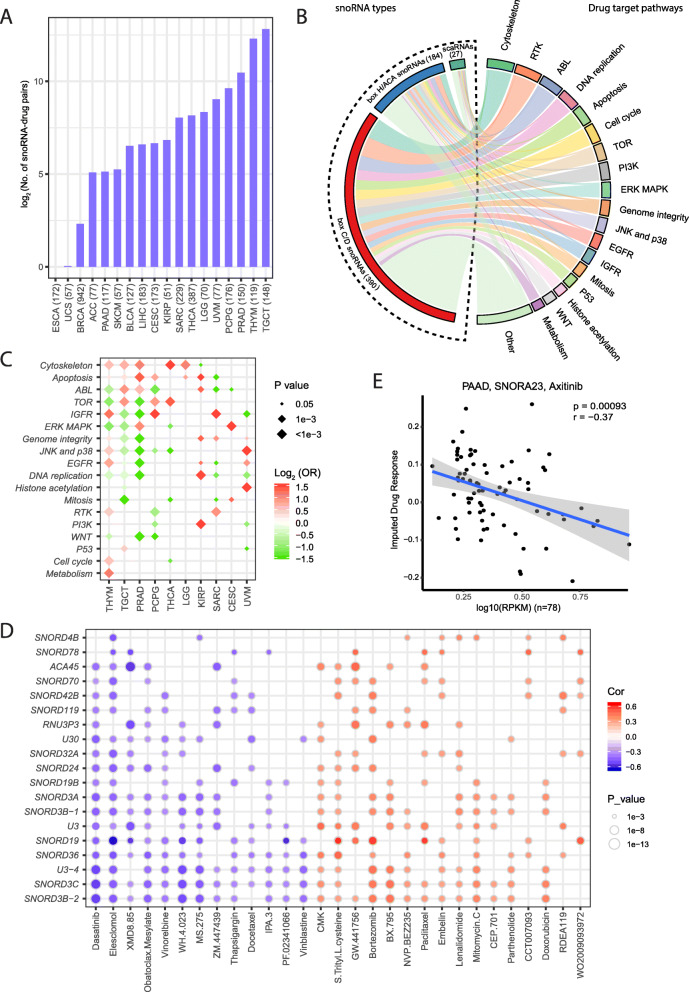
Pharmacogenomic landscape of snoRNAs. **a** Sample size included and significant snoRNA–drug pairs identified. **b** Association between snoRNA expression and imputed drug response. **c** Enrichment of various drug target pathways of significant snoRNA–drug response pairs. **d** Significantly correlated snoRNA–drug response pairs identified in PRAD. Drugs significantly associated with at least 5 snoRNAs are shown in the plot. **e** Association between SNORA23 expression and response to the drug axitinib in PAAD patients

To understand the effects of snoRNA expression on drug response, we performed an integrative analysis to assess the associations between the variance of snoRNA expression and the response to anticancer drugs in TCGA patients. We acquired imputed drug response data of TCGA patients from a previous study [9]. Eighteen cancer types in TCGA with at least 50 patients having both imputed drug response data and snoRNA expression data were included for drug response analysis. We identified 16,393 significantly correlated snoRNA–drug response pairs at FDR < 0.05 from 18 cancer types, ranging from 0 in esophageal carcinoma (ESCA) to 7226 in TGCT, with a median of 113 snoRNA–drug pairs per cancer type (Fig. 2a). The number of snoRNA–drug pairs did not correlate with the number of samples (Spearman’s correlation, *P*-value = 0.75). The snoRNAs (390 box C/D snoRNAs, 184 box H/ACA snoRNAs, and 27 scaRNAs) had extensive impact on patients’ responses to drugs within various drug target pathways across different cancer types. Among these drug target pathways, drugs related to the cytoskeleton pathway showed the largest number of drug–snoRNA pairs (Fig. 2b). We further used Fisher’s exact test to evaluate the enrichment of each drug target pathway in 10 cancer types with at least 100 significantly correlated snoRNA–drug response pairs identified, and found that the cytoskeleton pathway was significantly enriched in 5 cancer types (*P*-value < 0.05; Fig. 2c).

Taking prostate adenocarcinoma (PRAD) as an example, the response to 29 anti-cancer drugs significantly correlated with the levels of at least five snoRNAs (Fig. 2d). Intriguingly, various snoRNAs showed great consistency in having either a positive or negative effect on the response to one certain drug, which was likely due to the high correlation among the expression of these snoRNAs in each cancer type (additional file: Fig. S4). In patients with PAAD, the expression level of SNORA23 was highly associated with the response to the drug axitinib (R = − 0.37, *P*-value = 0.93 × 10^− 3^; Fig. 2e). SNORA23 has been shown to promote tumor growth and metastasis in pancreatic cancer [10]. Therefore, the expression level of SNORA23 may need to be considered in future clinical trials for axitinib. These results suggest that appreciable levels of snoRNAs could contribute to response to drug therapy. Future experiments are necessary to validate the effects of drug response for these snoRNAs.

### A comprehensive data resource to explore the genetic impacts and pharmacogenomic landscape of snoRNAs in cancer

**Fig. 3:**
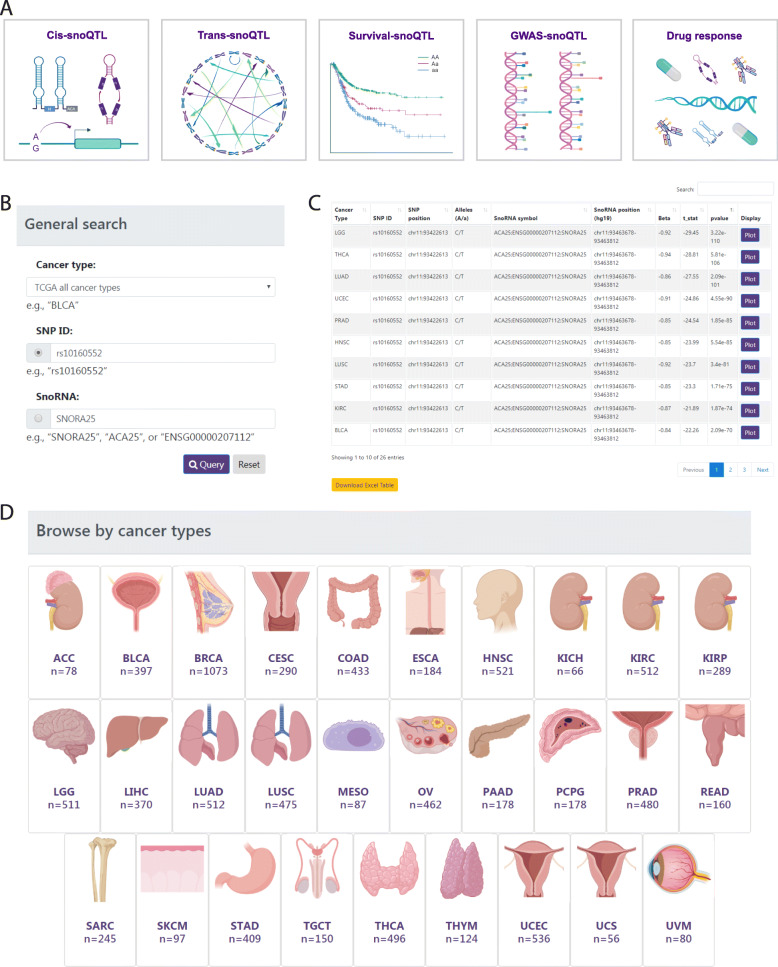
Web design and querying of GPSno. **a** Five main modules in GPSno. **b** General search section for querying. **c** Example of resulting list after querying on the cis/trans-snoQTL page. **d** Specific modules for querying results by cancer type

We developed a user-friendly data portal, GPSno (https://hanlab.uth.edu/GPSno/), to facilitate visualizing, searching and browsing of data by the biomedical research community. GPSno contains five main modules: *cis*-snoQTLs, *trans*-snoQTLs, survival-snoQTLs, GWAS-snoQTLs, and drug response (Fig. 3a). Several entryways are provided for querying, and each supports user-defined filters such as cancer type, SNP ID, and snoRNA ID. Users can enter different pages to search SNPs or snoRNAs of interest. We also provide a search section for users to query the data based on cancer type, SNP ID, or snoRNA (Fig. 3b). Querying on the *cis*/*trans*-snoQTL page, a table with SNP ID, SNP genomic position, SNP alleles, snoRNA ID, snoRNA position, beta value (effect size of SNP on gene expression), and *P*-value of snoQTL will be returned (Fig. 3c). For each record, a vector diagram of a boxplot is provided to display the association between SNP genotypes and snoRNA levels. Querying on the survival-snoQTL page, details with SNP ID, SNP genomic position, SNP alleles, log-rank test *P*-value and median survival times of different genotypes will be displayed. A vector diagram of the Kaplan–Meier plot is embedded in each record to display the association between snoQTL and overall survival times. Querying on the GWAS-snoQTLs page will return the SNP information, snoRNA information and related GWAS traits. Querying on the drug response page will return the snoRNA and related drug information, and a diagram is also provided to display the association between snoRNA levels and drug response. To facilitate researchers studying different cancer types, we also designed a cancer-type-specific module for querying results (Fig. 3d). Tables of querying results can be downloaded in XLSX format, and figures of results can be downloaded as a PDF. The document page includes an introduction, construction pipeline, and interpretation guidance for the database. This database is a valuable resource and will be of great interest to the research community, which will provide a unique resource to select candidate snoRNAs for future experiments.

## Conclusions

In light of the significance of snoRNAs in oncogenesis, we systematically investigated the impact of genetic variants and the pharmacogenomic landscape of snoRNAs in multiple cancer types from TCGA. We also developed GPSno as the first comprehensive data resource for the genetic and pharmacogenomic landscape of snoRNAs. Our study will shed light on future clinical considerations for the development of snoRNA-based targeted therapies.

## Supplementary information

**Additional file 1 Supplementary Figure 1.** (A) Workflow of snoQTL analysis. Blue boxes are software or packages utilized; green boxes are detailed modules or analysis used. Other boxes are input or output data in QTL analysis. (B) Relationship between snoQTLs identified and sample size included. **Supplementary Figure 2.** (A) Association between snoQTL rs1694419 alleles and SNORD45B levels in KIRC patients. (B) SNORD45B significantly upregulated in KIRC tumor tissues compared to adjacent normal tissues. (C) KIRC patients with higher expression of SNORD45B have worse overall survival than those with lower expression of SNORD45B. **Supplementary Figure 3.** Association between snoQTL rs12905354 alleles and SNORD18A levels in TGCT patients. **Supplementary Figure 4.** Co-expression of snoRNAs related to drug response found in PRAD.

**Additional file 2 Supplementary Table 1.** Abbreviations of cancer types. **Supplementary Table 2.** Analysis Summary

## Data Availability

The genotype and miRNAseq data of tumor patients can be found at the GDC portal (https://portal.gdc.cancer.gov/). The software and resources used in our analyses are described in each method section in the supplementary files. All results generated in this study can be found in our database, GPSno (http://hanlab.uth.edu/GPSno).

## Abbreviations

ACC: Adrenocortical carcinoma
BRCA: Breast invasive carcinoma
ESCA: Esophageal carcinoma
GWAS: Genome-wide association study
KIRC: Kidney renal clear cell carcinoma
LD: Linkage disequilibrium
LUAD: Lung adenocarcinoma
PAAD: Pancreatic adenocarcinoma
PRAD: Prostate adenocarcinoma
QTL: Quantitative trait loci
RPKM: Reads per kilobase per million reads
rRNA: Ribosomal RNA
snoQTL: snoRNA expression quantitative trait loci
snoRNA: Small nucleolar RNA
snRNA: Small nuclear RNA
TCGA: The Cancer Genome Atlas
THCA: Thyroid carcinoma
UCS: Uterine carcinosarcoma
