# Supplementary material for: The genetic and pharmacogenomic landscape of snoRNAs in human cancer
Source: Mol Cancer. 2020 Jun 23;19:108. doi: 10.1186/s12943-020-01228-z (PMC7313177; doi:10.1186/s12943-020-01228-z)
Supplement: Supplementary file 1 — Additional file 1 Supplementary Figure 1. (A) Workflow of snoQTL analysis. Blue boxes are software or packages utilized; green boxes are detailed modules or analysis used. Other boxes are input or output data in QTL analysis. (B) Relationship between snoQTLs identified and sample size included. Supplementary Figure 2. (A) Association between snoQTL rs1694419 alleles and SNORD45B levels in KIRC patients. (B) SNORD45B significantly upregulated in KIRC tumor tissues compared to adjacent normal tissues. (C) KIRC patients with higher expression of SNORD45B have worse overall survival than those with lower expression of SNORD45B. Supplementary Figure 3. Association between snoQTL rs12905354 alleles and SNORD18A levels in TGCT patients. Supplementary Figure 4. Co-expression of snoRNAs related to drug response found in PRAD. [file 12943_2020_1228_MOESM1_ESM.pdf]

A

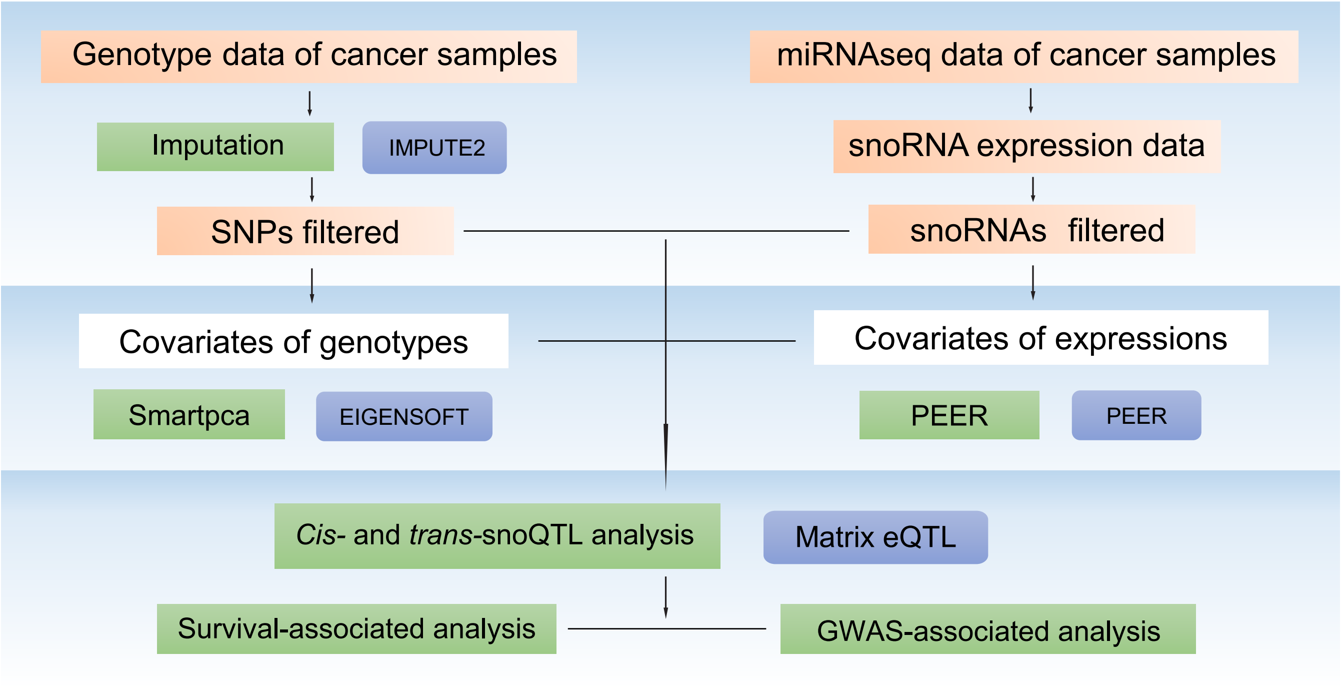

B

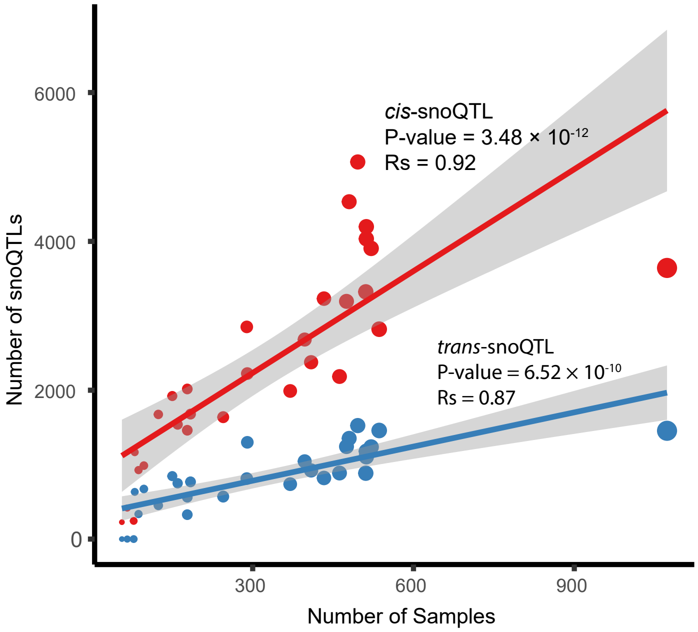

Supplementary Figure 1. (A) Workflow of snoQTL analysis. Blue boxes are software or packages utilized and green boxes are detailed modules or analysis used. Other boxes are input or output data in QTL analysis. (B) Relationship between snoQTLs identified and sample size included.

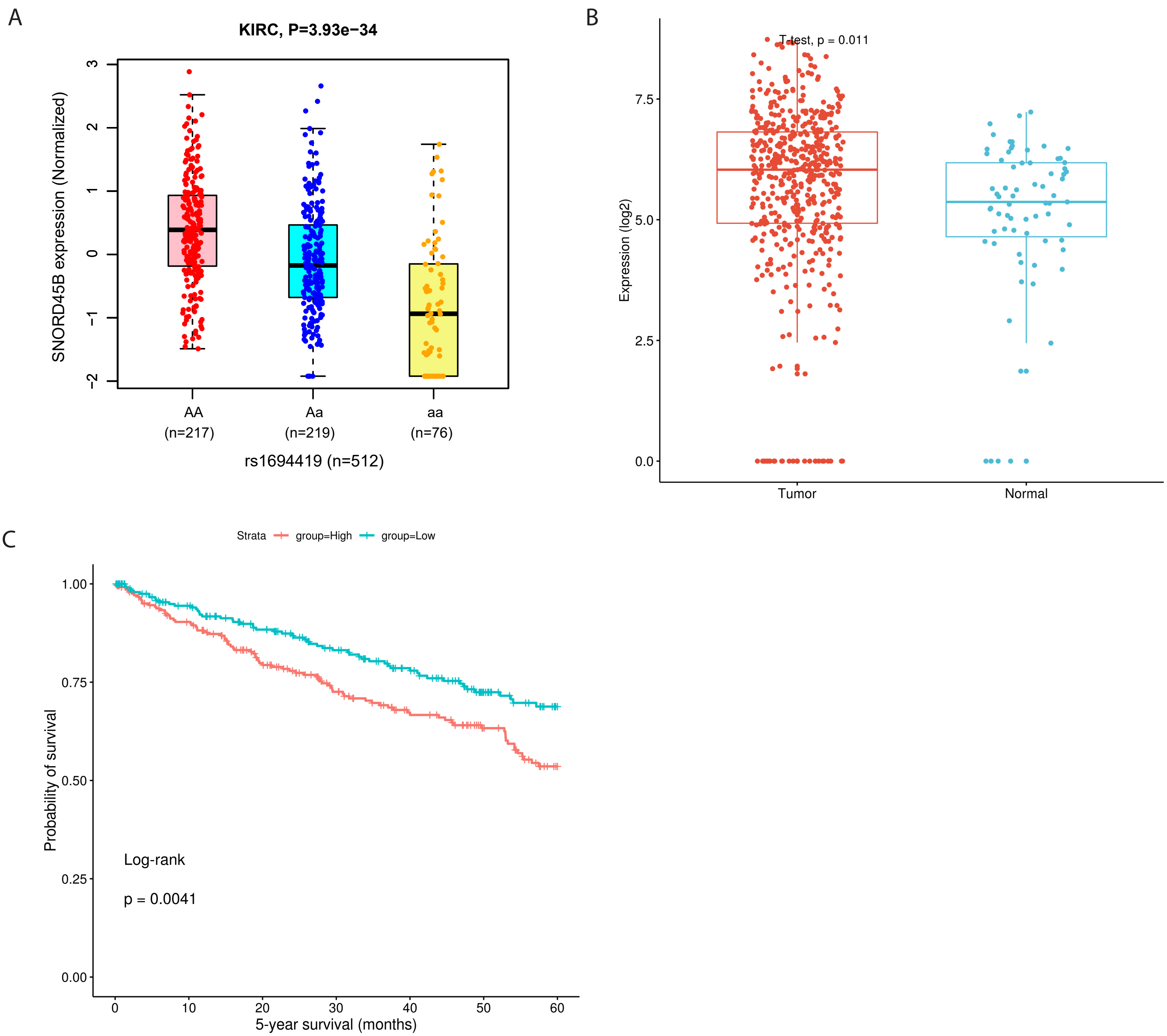

Supplementary Figure 2. (A) Association between snoQTL rs1694419 alleles and SNORD45B levels in KIRC patients. (B) SNORD45B significantly upregulated in KIRC tumor tissues compared to adjacent normal tissues. (C) KIRC patients with higher expression of SNORD45B have worse overall survival than those with lower expression of SNORD45B.

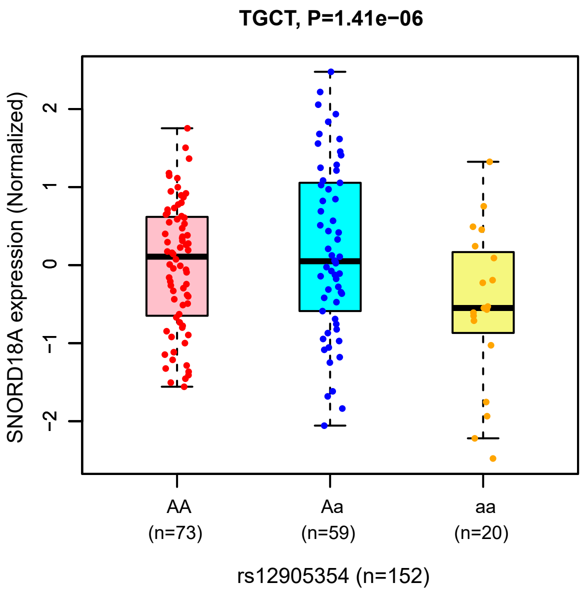

Supplementary Figure 3. Association between snoQTL rs12905354 alleles and SNORD18A levels in TGCT patients.

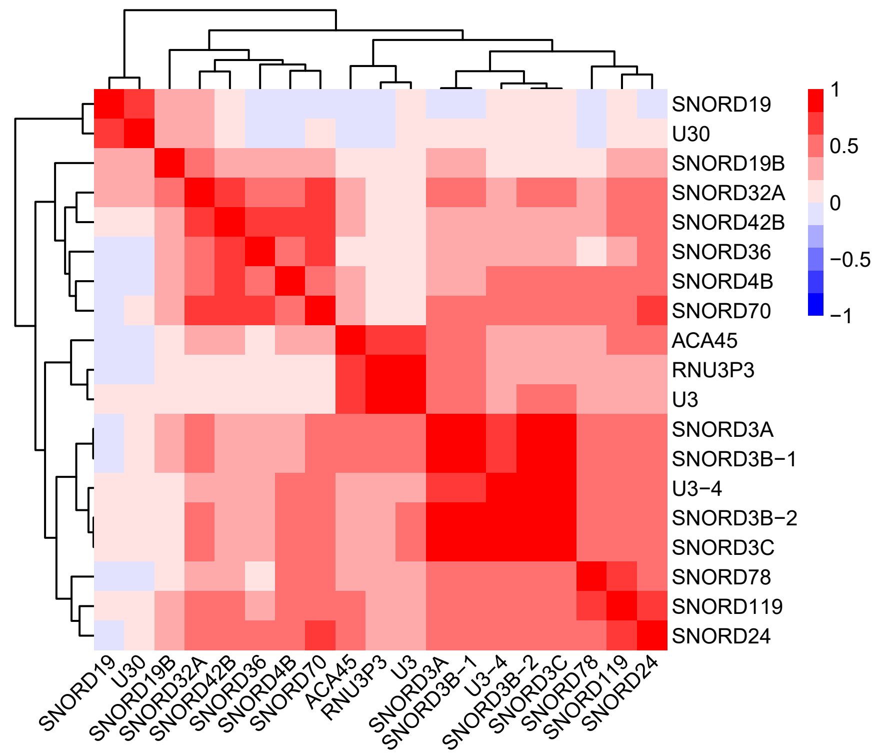

Supplementary Figure 4. Coexpression of snoRNAs relating drug response found in PRAD.
