# Supplementary material for: The genetic and pharmacogenomic landscape of snoRNAs in human cancer
Source: Mol Cancer. 2020 Jun 23;19:108. doi: 10.1186/s12943-020-01228-z (PMC7313177; doi:10.1186/s12943-020-01228-z)
Supplement: Supplementary file 2 — Additional file 2 Supplementary Table 1. Abbreviations of cancer types. Supplementary Table 2. Analysis Summary [file 12943_2020_1228_MOESM2_ESM.docx]

**SUPPLEMENTARY MATERIALS**

**The genetic and pharmacogenomic landscape of snoRNAs in human cancer**

**Materials and Methods**

**Supplementary Figures**

**Supplementary Tables**

**Supplementary References**

**Materials and Methods**

**Data collection and processing**

We downloaded genotype and miRNAseq data of tumor patients from TCGA data portal (<https://portal.gdc.cancer.gov/>). The SNP location (hg19 as reference) information was obtained from dbSNP (<https://www.ncbi.nlm.nih.gov/projects/SNP/>). For snoQTL analysis, we kept cancer types with tumor samples ≥ 50, and required the samples with both genotype and miRNAseq data. We obtained 9,449 samples across 29 cancer types. Imputation and quality filtering of genotype data were conducted as described in our protocol [1]. In brief, we imputed variants for all samples in each cancer type using IMPUTE2 [2], with 1000 Genomes Phase 3 as the reference panel [3]. After imputation, we used the following criteria to select SNPs [4]: (i) imputation confidence score, INFO ≥ 0.4, (ii) minor allele frequency (MAF) ≥ 5%, (iii) SNP missing rate <5% for best-guess genotypes at posterior probability ≥0.9, and (iv) Hardy–Weinberg equilibrium P-value > 1 × 10^−6^ estimated by Hardy–Weinberg R package [5]; we retained 4,358,817 genotypes per cancer type on average for further snoQTL analysis.

We obtained snoRNA expression profiling data from our previous study [6]. In brief, miRNAseq reads were mapped to snoRNA genes, and snoRNA expression was quantified as reads per kilobase per million reads (RPKM). SnoRNAs with an average RPKM ≥ 1 across samples in each cancer type were kept for further analysis. To diminish the effects of outliers on regression, the expression levels for each snoRNA across samples in each cancer type were transformed into a rank-based standard normal distribution [7]. We notated a snoRNA with a combination of its HUGO Gene Nomenclature Committee (HGNC) approved symbol, alias symbol, and/or ENSEMBL ID based on their availability, and prioritized the snoRNA ID in the order of HGNC symbol, alias symbol, and ENSEMBL ID.

We acquired imputed drug response data of TCGA patients from a previous study [8]. To assess the associations between the variance of snoRNA expression and drug response, we kept 18 cancer types in TCGA with at least 50 patients having both imputed drug response data and snoRNA expression data.

**Analysis of genetic impact on snoRNA expression**

To minimize the influence of confounders and increase sensitivity, we included covariates from the genotype and snoRNA data in the subsequent QTL analysis. The top five PCA factors attained from smartpca analysis in the EIGENSOFT program [9] were enrolled as covariates for the genotype data to control for ethnicity variance. To remove batch effects and other confounders in the snoRNA data, the top 15 PEER factors calculated by PEER [10] were chosen as covariates for snoRNA expression. Patient age, sex, and tumor stage were also included as common confounders. An average of ∼2 billion SNP–snoRNA pairs for each cancer type were analyzed in *cis*- and *trans*-snoQTL mapping by linear regression with adjusted covariates. Three N (genotype, expression or covariates) × S (samples) matrix files in matched sample order were acquired from the genotype data, snoQTL expression data and covariates for each cancer type. Matrix eQTL package [11] was used to perform linear regression analysis of effects of genetic variants on snoRNA expression. SNPs with false discovery rates (FDRs) <0.05 were defined as snoQTLs. If the position of a snoQTL was within 1 Mb from its related snoRNA, we defined it as *cis*-snoQTL; otherwise, it was regarded as *trans*-snoQTL. Clinical information of included samples was downloaded from TCGA data portal (<https://portal.gdc.cancer.gov/>). For each snoQTL, the log-rank test was applied to evaluate associations between snoQTL and overall survival times. Kaplan–Meier curves were generated to display individuals’ survival times for different genotypes. SnoQTLs with FDRs <0.05 were defined as survival-associated snoQTLs. We downloaded risk SNPs identified through genome-wide association study (GWAS) from the GWAS catalog (<http://www.ebi.ac.uk/gwas/>) [12]. GWAS linkage disequilibrium (LD) regions were extracted from SNAP [13] with the following parameters: SNP dataset: 1000 Genomes; population panel: CEU; r^2^ threshold: 0.5; distance limit: 500 kb. SnoQTLs that overlapped with GWAS tagSNPs and LD SNPs (r^2^ ≥0.5) were identified as GWAS-related snoQTLs.

**Pharmacogenomic analysis of snoRNAs**

To explore the impact of snoRNA expression on drug response, we acquired imputed drug response data of TCGA patients from a previous study [8]. Associations between standardized snoRNA expression and imputed drug response of cancer patients were analyzed using Pearson’s correlation. Drug response-associated snoRNAs were defined as those having FDRs <0.05. We used Fisher’s exact test to evaluate the enrichment of each drug target pathway in 10 cancer types with at least 100 significantly correlated snoRNA–drug response pairs identified.

**Database architecture**

The backend of the database, GPSno, was constructed under the R language-based SQL-like system, using the compressed R object and 'tidyverse' package suite. The frontend was built using Django framework (v1.9), Bootstrap (v4.0), and various JavaScript libraries. The five main modules were built in the database, and a general search section was provided for querying. Specific modules were designed for querying results by cancer type.

**Supplementary Figures**

**Supplementary Figure 1.** (A) Workflow of snoQTL analysis. Blue boxes are software or packages utilized; green boxes are detailed modules or analysis used. Other boxes are input or output data in QTL analysis. (B) Relationship between snoQTLs identified and sample size included.

**Supplementary Figure 2.** (A) Association between snoQTL rs1694419 alleles and SNORD45B levels in KIRC patients. (B) SNORD45B significantly upregulated in KIRC tumor tissues compared to adjacent normal tissues. (C) KIRC patients with higher expression of SNORD45B have worse overall survival than those with lower expression of SNORD45B.

**Supplementary Figure 3.** Association between snoQTL rs12905354 alleles and SNORD18A levels in TGCT patients.

**Supplementary Figure 4.** Co-expression of snoRNAs related to drug response found in PRAD.

**Supplementary Tables**

**Supplementary Table 1. Abbreviations of cancer types**

| ACC | Adrenocortical carcinoma |
| --- | --- |
| BLCA | Bladder urothelial carcinoma |
| BRCA | Breast invasive carcinoma |
| CESC | Cervical squamous cell carcinoma and endocervical adenocarcinoma |
| COAD | Colon adenocarcinoma |
| ESCA | Esophageal carcinoma |
| HNSC | Head and neck squamous cell carcinoma |
| KICH | Kidney chromophobe |
| KIRC | Kidney renal clear cell carcinoma |
| KIRP | Kidney renal papillary cell carcinoma |
| LGG | Lower grade glioma |
| LIHC | Liver hepatocellular carcinoma |
| LUAD | Lung adenocarcinoma |
| LUSC | Lung squamous cell carcinoma |
| MESO | Mesothelioma |
| OV | Ovarian serous cystadenocarcinoma |
| PAAD | Pancreatic adenocarcinoma |
| PCPG | Pheochromocytoma and paraganglioma |
| PRAD | Prostate adenocarcinoma |
| READ | Rectum adenocarcinoma |
| SARC | Sarcoma |
| SKCM | Skin cutaneous melanoma |
| STAD | Stomach adenocarcinoma |
| TGCT | Testicular germ cell tumors |
| THCA | Thyroid carcinoma |
| THYM | Thymoma |
| UCEC | Uterine corpus endometrial carcinoma |
| UCS | Uterine carcinosarcoma |
| UVM | Uveal melanoma |

| **Cancer type** | **No. of samples** | **No. of snoRNAs** | **No. of genotypes** | **Cis** | | **Trans** | | **Survival-associated snoQTLs** | **GWAS-associated snoQTLs** | **Drug response-related** | |
| --- | --- | --- | --- | --- | --- | --- | --- | --- | --- | --- | --- |
|  |  |  |  | **pairs** | **snoQTLs** | **pairs** | **snoQTLs** |  |  | **sample size** | **snoRNA-drug pairs** |
| ACC | 78 | 465 | 3 652 701 | 244 | 244 | 0 | 0 | 0 | 26 | 77 | 34 |
| BLCA | 397 | 411 | 4 221 213 | 2697 | 2681 | 1383 | 1045 | 48 | 853 | 127 | 92 |
| BRCA | 1073 | 464 | 2 791 786 | 3752 | 3643 | 2192 | 1455 | 2 | 982 | 942 | 5 |
| CESC | 290 | 400 | 4 417 705 | 2225 | 2224 | 1659 | 1300 | 88 | 716 | 173 | 101 |
| COAD | 433 | 456 | 4 191 023 | 3246 | 3231 | 1111 | 821 | 0 | 877 | 4 | - |
| ESCA | 184 | 401 | 4 541 112 | 1680 | 1679 | 1026 | 769 | 0 | 601 | 172 | 0 |
| HNSC | 521 | 415 | 4 311 502 | 4050 | 3906 | 1771 | 1237 | 0 | 1061 | 0 | - |
| KICH | 66 | 391 | 3 871 603 | 417 | 417 | 0 | 0 | 0 | 69 | 0 | - |
| KIRC | 512 | 441 | 4 600 464 | 4465 | 4197 | 1779 | 1180 | 36 | 1019 | 0 | - |
| KIRP | 289 | 424 | 4 948 299 | 2867 | 2851 | 1201 | 813 | 0 | 647 | 51 | 114 |
| LGG | 511 | 423 | 4 662 087 | 3333 | 3322 | 1251 | 885 | 2 | 973 | 70 | 324 |
| LIHC | 370 | 451 | 4 192 863 | 2059 | 1990 | 1066 | 738 | 6 | 621 | 183 | 97 |
| LUAD | 512 | 453 | 4 416 162 | 4308 | 4035 | 1679 | 1106 | 66 | 998 | 0 | - |
| LUSC | 475 | 446 | 3 811 695 | 3221 | 3194 | 1598 | 1243 | 0 | 927 | 0 | - |
| MESO | 87 | 414 | 4 861 983 | 927 | 927 | 441 | 336 | 0 | 233 | 0 | - |
| OV | 462 | 486 | 2 307 154 | 2299 | 2184 | 1509 | 887 | 11 | 583 | 0 | - |
| PAAD | 178 | 398 | 5 061 023 | 2017 | 2017 | 884 | 562 | 3 | 732 | 117 | 35 |
| PCPG | 178 | 401 | 4 804 919 | 1468 | 1462 | 377 | 328 | 27 | 287 | 176 | 791 |
| PRAD | 480 | 417 | 4 859 157 | 4555 | 4533 | 1970 | 1353 | 147 | 1048 | 150 | 1419 |
| READ | 160 | 472 | 4 638 399 | 1540 | 1539 | 1033 | 750 | 2 | 477 | 0 | - |
| SARC | 245 | 388 | 4 263 388 | 1641 | 1638 | 830 | 571 | 3 | 529 | 229 | 264 |
| SKCM | 97 | 451 | 4 768 644 | 987 | 987 | 958 | 673 | 14 | 331 | 57 | 38 |
| STAD | 409 | 434 | 4 406 785 | 2420 | 2376 | 1281 | 922 | 0 | 750 | 0 | - |
| TGCT | 150 | 472 | 4 893 554 | 1922 | 1922 | 1205 | 846 | 1 | 638 | 148 | 7226 |
| THCA | 496 | 435 | 4 889 302 | 5322 | 5067 | 2247 | 1525 | 8 | 1156 | 387 | 286 |
| THYM | 124 | 462 | 5 132 519 | 1675 | 1675 | 750 | 452 | 0 | 544 | 119 | 5043 |
| UCEC | 536 | 480 | 4 101 587 | 2826 | 2820 | 2079 | 1459 | 11 | 837 | 7 | - |
| UCS | 56 | 450 | 4 008 108 | 227 | 227 | 0 | 0 | 0 | 1 | 57 | 1 |
| UVM | 80 | 420 | 4 778 965 | 1167 | 1167 | 871 | 634 | 0 | 240 | 77 | 523 |

**Supplementary Table 2. Analysis Summary**

**Supplementary References**

1. Liu Y, Ye Y, Gong J, Han L. Expression Quantitative Trait Loci (eQTL) Analysis in Cancer. In: Xinghua S, editor. Methods Mol Biol. vol 2082. New York, NY: Humana; 2020. p. 189–99.

2. Howie BN, Donnelly P, Marchini J. A flexible and accurate genotype imputation method for the next generation of genome-wide association studies. PLoS Genet. 2009;5.

3. Auton A, Abecasis GR, Altshuler DM, Durbin RM, Bentley DR, Chakravarti A, et al. A global reference for human genetic variation. Nature. 2015. p. 68–74.

4. Ardlie KG, DeLuca DS, Segrè A V., Sullivan TJ, Young TR, Gelfand ET, et al. The Genotype-Tissue Expression (GTEx) pilot analysis: Multitissue gene regulation in humans. Science. 2015;348:648–60.

5. Graffelman J. Exploring diallelic genetic markers: The HardyWeinberg package. J Stat Softw. 2015;64:1–23.

6. Gong J, Li Y, Liu C jie, Xiang Y, Li C, Ye Y, et al. A Pan-cancer Analysis of the Expression and Clinical Relevance of Small Nucleolar RNAs in Human Cancer. Cell Rep. 2017;21:1968–81.

7. Ongen H, Andersen CL, Bramsen JB, Oster B, Rasmussen MH, Ferreira PG, et al. Putative cis-regulatory drivers in colorectal cancer. Nature. 2014;512:87–90.

8. Geeleher P, Zhang Z, Wang F, Gruener RF, Nath A, Morrison G, et al. Discovering novel pharmacogenomic biomarkers by imputing drug response in cancer patients from large genomics studies. Genome Res. 2017;27:1743–51.

9. Price AL, Patterson NJ, Plenge RM, Weinblatt ME, Shadick NA, Reich D. Principal components analysis corrects for stratification in genome-wide association studies. Nat Genet. 2006;38:904–9.

10. Stegle O, Parts L, Piipari M, Winn J, Durbin R. Using probabilistic estimation of expression residuals (PEER) to obtain increased power and interpretability of gene expression analyses. Nat Protoc. 2012;7:500–7.

11. Shabalin AA. Matrix eQTL: Ultra fast eQTL analysis via large matrix operations. Bioinformatics. 2012;28:1353–8.

12. Buniello A, Macarthur JAL, Cerezo M, Harris LW, Hayhurst J, Malangone C, et al. The NHGRI-EBI GWAS Catalog of published genome-wide association studies, targeted arrays and summary statistics 2019. Nucleic Acids Res. 2019;47:D1005–12.

13. Johnson AD, Handsaker RE, Pulit SL, Nizzari MM, O’Donnell CJ, De Bakker PIW. SNAP: A web-based tool for identification and annotation of proxy SNPs using HapMap. Bioinformatics. 2008;24:2938–9.
